# New Challenges Resulting From the Loss of Function of Na_v_1.4 in Neuromuscular Diseases

**DOI:** 10.3389/fphar.2021.751095

**Published:** 2021-10-04

**Authors:** Sophie Nicole, Philippe Lory

**Affiliations:** ^1^ Institut de Génomique Fonctionnelle (IGF), Université de Montpellier, CNRS, INSERM, Montpellier, France; ^2^ LabEx ‘Ion Channel Science and Therapeutics (ICST), Montpellier, France

**Keywords:** sodium channel, skeletal muscle, loss of function, therapeutics, congenital myasthenic syndrome (CMS), congenital myopathy (CM)

## Abstract

The voltage-gated sodium channel Na_v_1.4 is a major actor in the excitability of skeletal myofibers, driving the muscle force in response to nerve stimulation. Supporting further this key role, mutations in *SCN4A*, the gene encoding the pore-forming α subunit of Na_v_1.4, are responsible for a clinical spectrum of human diseases ranging from muscle stiffness (sodium channel myotonia, SCM) to muscle weakness. For years, only dominantly-inherited diseases resulting from Na_v_1.4 gain of function (GoF) were known, *i.e.*, non-dystrophic myotonia (delayed muscle relaxation due to myofiber hyperexcitability), *paramyotonia congenita* and hyperkalemic or hypokalemic periodic paralyses (episodic flaccid muscle weakness due to transient myofiber hypoexcitability). These last 5 years, *SCN4A* mutations inducing Na_v_1.4 loss of function (LoF) were identified as the cause of dominantly and recessively-inherited disorders with muscle weakness: periodic paralyses with hypokalemic attacks, congenital myasthenic syndromes and congenital myopathies. We propose to name this clinical spectrum sodium channel weakness (SCW) as the mirror of SCM. Na_v_1.4 LoF as a cause of permanent muscle weakness was quite unexpected as the Na^+^ current density in the sarcolemma is large, securing the ability to generate and propagate muscle action potentials. The properties of *SCN4A* LoF mutations are well documented at the channel level in cellular electrophysiological studies However, much less is known about the functional consequences of Na_v_1.4 LoF in skeletal myofibers with no available pertinent cell or animal models. Regarding the therapeutic issues for Na_v_1.4 channelopathies, former efforts were aimed at developing subtype-selective Na_v_ channel antagonists to block myofiber hyperexcitability. Non-selective, Na_v_ channel blockers are clinically efficient in SCM and *paramyotonia congenita*, whereas patient education and carbonic anhydrase inhibitors are helpful to prevent attacks in periodic paralyses. Developing therapeutic tools able to counteract Na_v_1.4 LoF in skeletal muscles is then a new challenge in the field of Na_v_ channelopathies. Here, we review the current knowledge regarding Na_v_1.4 LoF and discuss the possible therapeutic strategies to be developed in order to improve muscle force in SCW.

## Introduction

**FIGURE 1:**
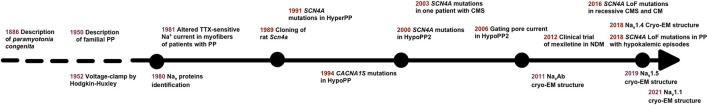
Timeline highlighting important events for Na_v_1.4 channelopathies. PP: periodic paralysis; TTX: tetrodotoxin; HyperPP, hyperkalemic PP; HypoPP, hypokalemic PP; HypoPP2, hypokalemic PP, type 2; CMS, congenital myasthenic syndrome; cryo-EM, cryo-electron microscopy; NDM, non-dystrophic myotonia; LoF, loss of function; CM, congenital myopathy.

Voltage-gated sodium (Na^+^) channels (Na_v_) initiate and conduct action potentials (AP) in excitable cells in response to membrane depolarization. The first Na^+^ channelopathy identified in humans was the hyperkalemic form of periodic paralysis (HYPP or HyperPP, OMIM#170500) 30 years ago (Figure 1): missense mutations in *SCN4A*, the gene encoding the pore-forming subunit of Na_v_1.4 channels, were reported as the cause of this familial form of transient muscle weakness. This pioneer demonstration was done using a combination of patch-clamp recordings from HyperPP myotubes, genetic linkage in HyperPP families and screening for mutations in *SCN4A* (Lehmann-Horn et al., 1983; Fontaine et al., 1990; Ptáček et al., 1991). Strengthening its prototypical role for Na_v_ channels, Na_v_1.4 is also the first human Na_v_ channel for which the 3D structure has been determined by cryo-electron microscopy (cryoEM) at the atomic resolution (Pan X. et al., 2018). Gain of Function (GoF) of Na_v_1.4 (*i.e.*, an overactive channel) is now known to cause a spectrum of three clinically delineated dominantly-inherited neuromuscular disorders with overlapping clinical symptoms: sodium channel myotonia (SCM), *paramyotonia congenita* (PMC) and primary periodic paralyses (PP) (Cannon, 2018). They span a continuum of altered membrane excitability and form the group of muscle Na^+^ channelopathies, which are ultra-rare diseases with a prevalence estimated to be around 1–2/100,000 (Horga et al., 2013; Stunnenberg et al., 2018a). More than 70 GoF mutations in *SCN4A*, all missense, have been reported in these diseases (Cannon, 2018; Maggi et al., 2021). A few are *de novo,* especially those causing neonatal forms of life-threatening myotonia (severe neonatal episodic laryngospasm or SNEL, *myotonia permanens*) if not treated with Na_v_ blockers (Lion-Francois et al., 2010; Lehmann-Horn et al., 2017).

The properties of *SCN4A* GoF mutations on Na_v_1.4 gating behavior have been well studied using heterologous cell expression systems, mouse models and computer simulations, providing the community with pathophysiological mechanisms. Exhaustive structure-function aspects of Na_v_1.4 channels, Na_v_1.4 GoF mutations and related channelopathies are well discussed in recent reviews and are not the scope here (Cannon, 2018; Catterall et al., 2020; Maggi et al., 2021; Mantegazza et al., 2021; Meisler et al., 2021). Briefly, all GoF missense mutations except those resulting in hypokalemic periodic paralyses (HOKPP or HypoPP, type 2 OMIM**#** 613,345) enhance activation or impair fast inactivation of Na_v_1.4. That increases Na^+^ influx in the myofibers, causes repetitive APs and delays muscle relaxation in SCM and PMC. Na_v_1.4 GoF in HyperPP leads to sustained membrane depolarization, inactivation of Na_v_1.4 channels and unresponsiveness of myofibers. A distinct mechanism causes the familial forms of HypoPP: the missense mutations favor an inward rectifying cation current through a gating pore that leads to sustained membrane depolarization. The HypoPP2 mutations exert also LoF effects on Na_v_1.4 gating with unknown physiological impact.

Recessively-inherited hypomorph (reduced function) or null (no function at all) mutations that cause Na_v_1.4 LoF and muscle weakness have been reported just recently, 25 years after the report of the first *SCN4A* mutation in HyperPP (Figure 1). These recent works have underlined the lack of efficient therapeutic solutions against Na_v_1.4 LoF and have drawn attention back to Na_v_1.4 LoF in HypoPP2. In this review, we describe some molecular aspects of Na_v_1.4 in skeletal muscles, present the Na_v_1.4 LoF mutations and discuss possible therapeutic strategies to counteract their deleterious effect on muscle force.

## Na_v_ and Skeletal Muscles

### A Brief Overview of Na_v_ Structure-Function

**FIGURE 2:**
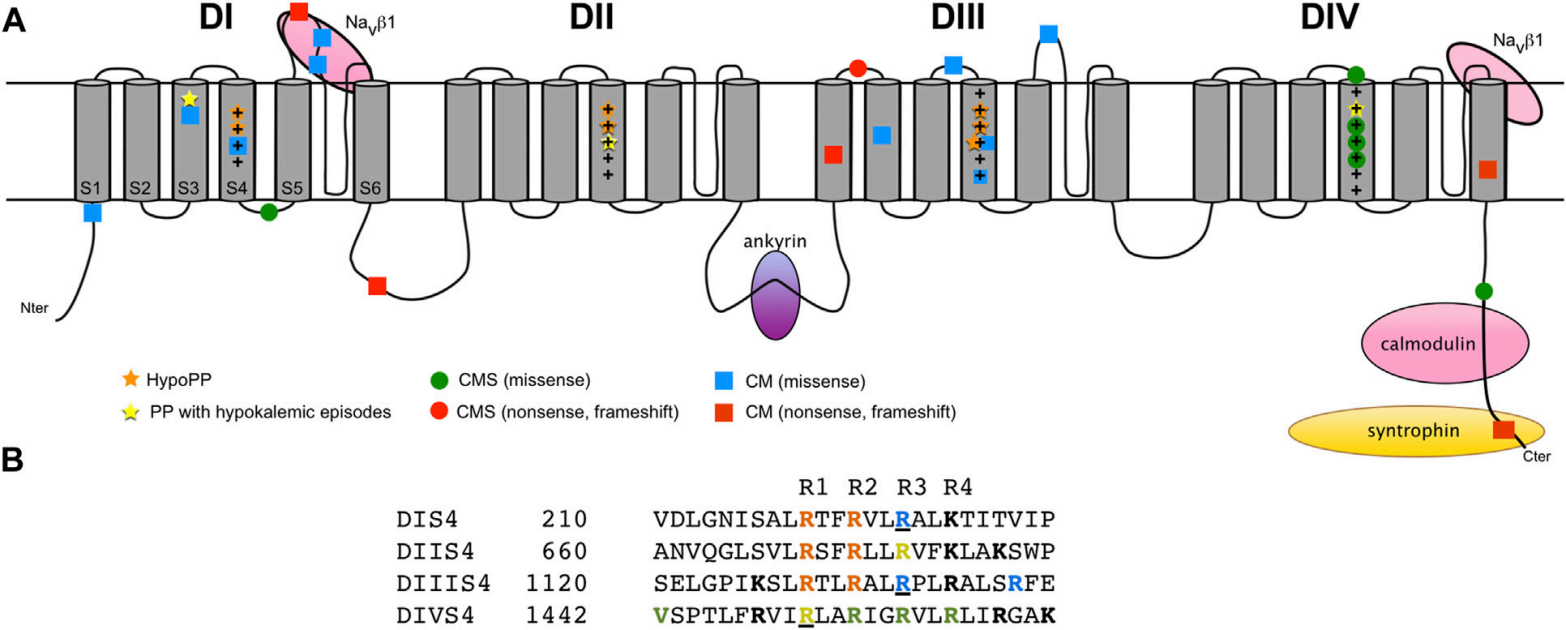
Structure of the pore-forming α subunit of human Na_v_1.4 and localization of the *SCN4A* loss-of-function (LoF) mutations in monogenic human disorders. **(A)** Schematic membrane topology of Na_v_1.4 α subunit with four domains (DI-DIV), each domain being composed of six transmembrane segments (S1-S6). The voltage-sensor S4 segments are rich in positively-charged amino acid residues (+). The LoF mutations are shown. Hypokalemic periodic paralysis (HypoPP), orange stars; periodic paralysis (PP) with hypokalemic episodes, yellow stars; CMS, green (missense mutation) and red (nonsense or frameshift mutation) circles; CM, blue (missense mutation) and red (nonsense or frameshift mutation) squares. The sites of binding interactions with the transmembrane β1 subunit, and the cytoplasmic ankyrin, calmodulin and syntrophin proteins are indicated. **(B)** Alignment of amino acid sequences of the four S4 segments of human Na_v_1.4 with positively-charged residues (in bold). The gating charges are named R1 to R4. The position of the missense mutations causing HypoPP2 (orange), PP with hypokalemic episodes (yellow), CMS (green) and CM (blue) is indicated. Underlined residues cause different phenotypes when substituted: dominant NDM or recessive CM (p.Arg225); *de novo* HypoPP2, recessive HypoPP2 or CM (p.Arg1135); dominant NDM, glucocorticoid-induced HypoPP, and dominant or recessive PP with hypokalemic episodes (p.Arg1451). To note that the substitutions of p.Arg675 (R3, DIIS4) and p.Arg1135 (R3, DIIIS4) cause HypoPP2 or normokalemic PP with corticosteroid- or thyrotoxicosis-induced hypokalemic episodes of paralysis and depolarization- and not hyperpolarization-activated gating pore current (Vicart et al., 2004; Sokolov et al., 2008; Groome et al., 2014).

Na_v_ channels are composed of one large, pore-forming α subunit with one or two auxiliary β subunits. Nine Na_v_ isoforms (Na_v_1.1-Na_v_1.9) are described in mammals. Each isoform is characterized by its electrophysiological and pharmacological properties, as well as its tissue expression pattern. An additional and atypical Na^+^ channel isoform, Na_x_, is not voltage-dependent and arises as a different Na^+^ channel subfamily (Dolivo et al., 2021). The pore-forming α subunit of Na_v_ forms a functional Na^+^ channel and the β subunits modulate its trafficking and electrophysiological properties. The α subunit is organized in four homologous transmembrane repeat domains (DI-DIV). Each domain contains six transmembrane segments (S1-S6) that are divided into two main functional modules: the voltage-sensing domain (S1-S4) and the pore module (S5-S6) (Figure 2). The β subunits are multifunctional glycoproteins with one single transmembrane segment, and four isoforms exist in mammals (β1-β4 encoded by *SCN1B*-*SCN4B*) (Winters and Isom, 2016).

Na_v_ channels have three main distinct conformational states: resting (closed), activated (open), inactivated (closed). The kinetics of each transition, *i.e.* activation (from resting to activated), inactivation (from activated to inactivated) and recovery from inactivation (from inactivated to resting) shape the electrophysiological behavior of Na_v_ channels and critically determine AP frequency. Inactivation includes two distinct components: fast-inactivation and slow-inactivation, fast-inactivation being the main feature of Na_v_ channels. Na_v_ channels activate rapidly (in less than 1 ms) in response to membrane depolarization. The major structural determinants in this process are the S4 transmembrane segments. They are composed of positively charged arginine (or lysine) residues, also called gating charges, occurring in a Arg/Lys-X-X repetitive sequence (Figure 2). Upon depolarization, the S4 helices undergo outward movements by electrostatic interactions of their basic residues with conserved acidic or polar residues of S2 and S3 segments (Catterall et al., 2020). This outward movement drives the conformational shifts of the channel in response to depolarization with opening of the central (also known as α) pore formed by the P-loops of the four S5–S6 segments. The open central pore then conducts a large inward Na^+^ current that drives the depolarization phase of AP. Within a few milliseconds following their activation, Na_v_ channels undergo fast inactivation, which facilitates membrane repolarization. Fast inactivation is mainly dictated by the movement of DIVS4 during activation (Capes et al., 2013). During this conformational change, the inactivation gate formed by the cytoplasmic linker between DIII and DIV intracellularly blocks the central pore. Prolonged or high-frequency depolarizations drive Na_v_ channels into a slow-inactivated state with time constant ranging from 100 ms to several minutes. Slow inactivation determines long-lasting Na_v_ channels availability, thereby defining the cell firing properties. Its structural basis remains unclear. It probably relies on several sequential conformational changes including constriction of the pore by rearrangement of the ion selectivity filter and S6 segments (Mantegazza et al., 2021).

### Na_v_1.4 Channels and Myofiber Action Potential

**FIGURE 3:**
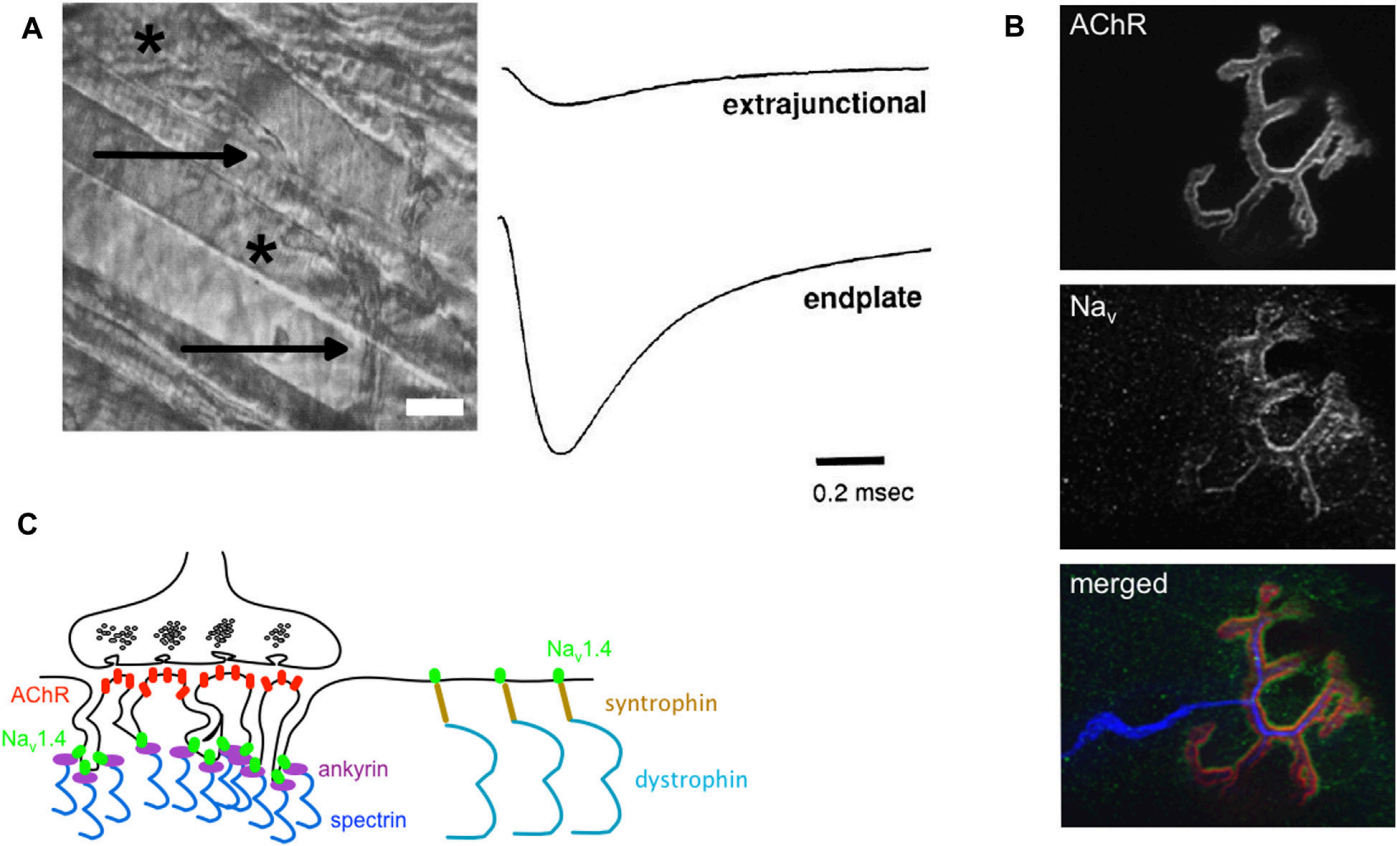
Na_v_ channels, Na^+^ current and neuromuscular junctions. **(A)** Left: a phase-contrast image of an adult mouse *Levator aureus longus* (LAL, fast-twitch) muscle. The myelinated nerve (arrows) terminates at neuromuscular junctions (NMJs, stars) (scale bar, 15 μm). Right: examples of Na^+^ current recordings made with a loose-patch clamp electrode from the extrasynaptic membrane (extrajunctional, top trace) and synaptic (endplate, bottom trace) membrane of a LAL myofiber. The extrasynaptic Na^+^ current density is equal to 23.3 mA/cm^2^ and the endplate Na^+^ current density is equal to 110 mA/cm^2^ (adapted from (Lupa et al., 1993); Copyright [1993] Society for Neuroscience). **(B)** Confocal image of fluorescent staining of nAChRs (stained with α bungarotoxin, in red in the merged image), Na_v_ channels (anti-pan antibody binding to all Na_v_ isoforms, in green in the merged image), and nerve (anti-NF 200 and anti-synaptophysin antibodies, in blue in the merged image) on dilacerated *Tibialis anterior* (fast-twitch) myofibers from an adult mouse (X 63). **(C)** Schema of one NMJ with its presynaptic (nerve terminal rich in synaptic vesicles, up) and post-synaptic (myofibers with post-synaptic folds, down). AChRs (red) are clustered at the top and Na_v_ channels (Na_v_1.4 in green) are clustered in the depth of the postsynaptic folds. The synaptic aggregation of Na_v_1.4 would result from its binding interaction with ankyrins (purple), themselves linked to spectrin (deep blue). Na_v_1.4 would interact with syntrophin (brown) in the extrasynaptic sarcolemma.

The force developed by a muscle from a single twitch to its maximal sustained contraction (known as tetanus) resulting from the twitch summation relies on the AP frequency, and by this way on the functional properties of Na_v_1.4. In the adult myofiber, the AP is generated at the neuromuscular junction (NMJ) located in the middle of the myofiber when the endplate potential (epp), a local depolarization induced by the opening of the post-synaptic nicotinic acetylcholine receptors (nAChRs), reaches the threshold (-50 mV) for AP genesis. To decrease the effective threshold for AP generation and favor the latter, the amplitude of Na_v_ currents is higher at the NMJ than in the extrasynaptic area (80 mA/cm^2^ against 20 mA/cm^2^, adult fast-twitch myofibers of mouse *Levator auris longus*) (Figure 3) (Lupa et al., 1993; Slater, 2003). The high synaptic Na^+^ current density results from the clustering of Na_v_ channels at the NMJ, more specifically in the depth of the postsynaptic folds while the tops of the folds are enriched in AChRs (Figure 3). The AP generated at the NMJ then propagates longitudinally toward the extremities and radially through the transverse tubules to drive excitation-contraction coupling and myofiber contraction. In the extrasynaptic area, the distribution of Na_v_ channels between the “surface” and transverse tubules sarcolemma is estimated to be in the range between 40:60 and 60:40 (DiFranco and Vergara, 2011).

Two Na_v_ currents exist in skeletal myofibers. In innervated adult myofibers of rodents, a tetrodotoxin (TTX)-sensitive Na^+^ current (IC_50_ 5 nM) generated by Na_v_1.4 (formerly SkM1) accounts for 98% of the whole inward Na^+^ current (Fu et al., 2011). In immature neonatal myofibers, this current is low or null compared to a TTX-resistant Na^+^ current (SkM2, IC_50_ 2000 nM). The TTX-resistant current is mediated by Na_v_1.5 encoded by *SCN5A*, which is the main cardiac Na_v_ isoform (Loussouarn et al., 2015). In addition to distinct TTX sensitivity, these two Na^+^ currents have different electrophysiological properties with higher activation time constants and lower conductance for Na_v_1.5 current compared to Na_v_1.4 current (Morel et al., 2010). The switch between Na_v_1.5 and Na_v_1.4 occurs between birth and the third postnatal week in rodents and is concomitant to the maturation of NMJs and myofibers (Lupa et al., 1993). The neonatal lethality of children and mice with bi-allelic null mutations in *SCN4A* demonstrates that Na_v_1.5 does not compensate for the lack of Na_v_1.4 in skeletal muscles at birth (Wu et al., 2016; Zaharieva et al., 2016).

Not all myofibers are equal regarding excitability and Na_v_ current properties. Slow-twitch motor units in mammals are active at 10–20 Hz and fast-twitch motor units are active at rates from 40 to 90 Hz to more than 200 Hz for brief periods (Hennig and Lømo, 1985). To assume these high frequencies, the Na^+^ current density is two-to six-fold higher in fast-than in slow-twitch myofibers (Ruff, 1996; Desaphy et al., 2001). The difference in synaptic *versus* extrasynaptic Na^+^ current density is also more important in fast-twitch compared to slow-twitch myofibers (Milton et al., 1992). Slow inactivation of Na^+^ current is less prominent in slow-twitch fibers (Ruff, 1996). These distinct characteristics probably play a role in the excitability and contractility properties of myofibers such as threshold potential, time to reach contraction threshold or resistance to fatigue.

It is here important to note inter-species differences. For example, the time constants for entry or recovery from slow inactivation are reported to be 4 times faster in human than in rat myofibers (Ruff, 1996). Individual human muscles tend to be a mixture of various fiber types. Whether and when the Na_v_1.5/Na_v_1.4 switch occurs in humans is not documented. Human NMJs are smaller and have more extensive postsynaptic folds than rodent NMJs, which may have functional significance since the NMJ size and the spacing and depth of the postsynaptic folds have an important effect on AP genesis (Jones et al., 2017; Slater, 2017). Moreover, proteomics analyses have recently provided evidence that human NMJs have a significantly modified molecular composition compared to mouse NMJs (Jones et al., 2017). These differences must be taken into consideration when interpreting animal-based studies with respect to their applicability to humans.

### Molecular Interactions for Na_v_1.4

Protein interactions of Na_v_ channels modulate their trafficking, cellular localization and functional properties. Little is known about these important functional aspects for Na_v_1.4 compared to other Na_v_ channels (Loussouarn et al., 2015; Eshed-Eisenbach and Peles, 2020). If sequence similarities suggest the possibility that Na_v_1.4 shares binding partners and regulatory mechanisms with other Na_v_ isoforms, further investigations are required to prove this assumption and to best understand the fine-tuning of Na_v_1.4 in skeletal myofibers.

One regulatory β subunit, mainly β1 (encoded by *SCN1B*), associates non-covalently with the Na_v_1.4 α subunit (Yang et al., 1993). The β2 (*SCN2B*), β3 (*SCN3B*) or β4 (*SCN4B*) subunits might also contribute to the Na_v_1.4 channel complex since their transcripts are detected in muscle cells (David et al., 2008; Morel et al., 2010). Two extracellular binding sites (DIS5S6 and DIVS5S6) of Na_v_1.4 are involved in the interaction with β1 (Figure 2) (Makita et al., 1996). Different effects of the interaction of αNa_v_1.4 with β1 on Na^+^ current have been reported depending on the heterologous cell expression system. They include increased current density, enhanced inactivation with faster kinetics and hyperpolarization shift (Cannon et al., 1993). The differences between cell expression systems may result from distinct β1 glycosylation, which would modify Na_v_ channel gating through the density of the surrounding surface charges (Ferrera and Moran, 2006). No mutations in the genes encoding β subunits are reported to cause disorders with altered skeletal muscle force (Winters and Isom, 2016). It remains to be determined whether this is due to a lower sensitivity of Na_v_1.4 to functional modulation by β subunits in myofibers compared to other Na_v_ isoforms, to compensation between β isoforms or to the subjective nature of mild variations in muscle force, which may not be considered as “pathogenic” by individuals suffering from them.

The mechanisms leading to the synaptic accumulation of Na_v_1.4 are still unclear compared to those resulting in the clustering of neuronal channels at axonal initial segments or nodes of Ranvier (Eshed-Eisenbach and Peles, 2020). Synaptic aggregation of Na_v_1.4 channels would result from their binding interaction with ankyrins (Figure 3). Ankyrins are scaffolding proteins that link membrane-bound proteins to the underlying spectrin-actin cytoskeleton. Ankyrins have a pivotal role in the anchorage of Na_v_ channels to the submembranous cytoskeleton through their binding interaction with a motif of 27 amino acid residues located within the cytoplasmic DII-DIII linker of Na_v_ channels (Figure 2) (Garrido et al., 2003). A recent work has confirmed that ankyrins are required to cluster Na_v_ channels in the depth of synaptic folds since the lack of ankyrins G, R and B induced loss of Na_v_ staining at the NMJ (Zhang et al., 2021). The clustering of Na_v_ channels in the depth of the folds is supposed to result from the physical exclusion of the ankyrin-Na_v_1.4 complexes from the tops rich in nAChRs during NMJ formation maturation (Flucher and Daniels, 1989; Bailey et al., 2003).

Na_v_1.4 also interacts with α syntrophin, a peripheral membrane protein localized to the cytosolic face that links a variety of signaling proteins and ion channels to the dystrophin-associated protein complex (Figure 3) (Gee et al., 1998). The C-terminal tail of Na_v_1.4 possesses a SerLeuVal peptidic sequence that binds the PDZ (for « PSD-95, Dlg1, Zo-1 ») domain of syntrophin (Figure 2). The synaptic immunostaining of syntrophin is distinct from Na_v_ immunostaining at the NMJ, suggesting that their interaction solely takes place in the extrasynaptic region (Bailey et al., 2003). The syntrophin-Na_v_1.4 interaction could modify the gating properties of Na_v_1.4 and participate to loss of muscle force in some acquired or inherited myopathic conditions, such as critical illness myopathy or Duchenne muscular dystrophy, a disease acquired by patients in intensive care units and an inherited disorder characterized by progressive muscle degeneration and weakness due to the lack of dystrophin, respectively (Teener and Rich, 2006; Hirn et al., 2008; Kraner et al., 2012).

Calmodulin is one of the rare proteins well known to modulate Na_v_1.4 gating (Nathan et al., 2021). This intracellular multifunctional protein is ubiquitously expressed and acts as part of a Ca^2+^ signal transduction pathway by modifying its interactions when bound to Ca^2+^. Calmodulin interacts with an IQ domain downstream of an EF-hand domain located in the cytoplasmic C-terminal tail of Na_v_1.4 (Figure 2). The interaction of Na_v_1.4 with calmodulin facilitates its cell surface expression and mediates its Ca^2+^-dependent slow inactivation (Biswas et al., 2008; Yoder et al., 2019). The crystal structure of Ca^2+^-calmodulin bound to Na_v_1.4 suggests that the Ca^2+^-dependent inactivation of Na_v_1.4 would result from the relative reorientation of its EF-hand and IQ domains (Gardill et al., 2019). Increasing the intracellular pool of Ca^2+^ released from the sarcoplasmic reticulum inhibits Na^+^ currents in mouse skeletal muscles (Sarbjit-Singh et al., 2020; Liu et al., 2021). These data suggest activity-dependent feedback mechanisms of Na_v_1.4 activity by intracellular Ca^2+^ released from the transverse tubules for a fine tuning of muscle contraction during periods of repetitive activity.

To complete this list of Na_v_1.4 binding interaction, it is worth noting that the α subunits of Na_v_1.5, Na_v_1.1, Na_v_1.2 and Na_v_1.7 form functional dimers (Clatot et al., 2017; Rühlmann et al., 2020). The dimerization involves a 20 amino acid motif within the DI-DII cytoplasmic loop and is mediated by the 14-3-3 protein. Na_v_1.4 is the sole Na_v_ channel lacking this motif, suggesting that functional dimerization does not occur for this isoform (Rühlmann et al., 2020).

### *SCN4A* Gene Expression

The human *SCN4A* gene (24 exons, chromosome 17q23.3) codes for a single mRNA transcript transcribed into a protein of 1,836 amino acids (208 kDa). *SCN4A* is referred to as the skeletal muscle isoform since its cDNA cloning 30 years ago (Trimmer et al., 1989). A small expression of *SCN4A* in heart tissue and cardiac myocytes is reported (1.1% of relative Na_v_ channels transcript levels in humans) (Blechschmidt et al., 2008; Kaufmann et al., 2013). The tissue-specific expression of *SCN4A* most probably explains why Na_v_1.4 dysfunction only impacts skeletal muscles even if anecdotic reports of cardiac arrhythmia in individuals with dominant *SCN4A* mutations have been published (Loussouarn et al., 2015).

In rodents, *Scn4a* expression is developmentally regulated. The amount of *Scn4a* transcripts increases from birth to 2 weeks after birth while *Scn5a* (the gene encoding the pore-forming subunit of Na_v_1.5) expression decreases (Lupa et al., 1993). This developmental period is concomitant to the maturation of the neuromuscular system, especially the NMJ, in rodents (Li et al., 2018). The high density of Na_v_ channels at the NMJ would result in part from the accumulation of *Scn4a* mRNA at the synapse (Lupa et al., 1993; Awad et al., 2001; Bailey et al., 2003). However, *Scn4a* expression is insensitive to denervation in the adult myofiber, suggesting that its synaptic expression is not strictly regulated by the nerve terminal (Lupa and Caldwell, 1991; Awad et al., 2001; Carreras et al., 2021). Northern blot analyses have showed that *Scn1b* expression parallels *Scn4a* expression during postnatal development and after denervation in rodents, suggesting that common factors regulate their gene expression (Yang et al., 1993). Whether similar modulations of *SCN4A* and *SCN1B* gene expression with NMJ development and denervation exist in humans has not been studied.

The specific expression of *Scn4a* in differentiated myofibers is controlled by at least 4 *cis*-regulatory promotor elements working together: 1) a core promoter that lacks muscle specificity; 2) a repressor that confers muscle specificity; 3) a muscle-specific positive element, whose accessibility is partially masked by the repressor; and 4) another muscle-specific positive element that confers a 10-fold up-regulation of expression (Hebert et al., 2007; Kraner et al., 1999; Kraner et al., 1998). The cumulative binding of non-specific (Nuclear Factors (NF) I, ZEB/AREB6 and REB) and muscle-specific (myogenin and MRF4) transcription factors on these elements would contribute to the upregulation of *Scn4a* expression in myofibers. According to the fact that denervation does not modify its expression, no N-box—a response element that promotes synaptic-specific expression in response to the neuronal factors agrin and neuregulins for NMJ formation and maturation (Belotti and Schaeffer, 2020) — is present within the promotor of *Scn4a*, suggesting the involvement of other regulatory mechanisms. The simplest hypothesis is a higher synaptic amount of *Scn4a* mRNA due to the high number of myonuclei in this area.

Several transcripts exist for *SCN5A* but none is specific to skeletal myofibers. Na_v_1.5 GoF or LoF due to *SCN5A* mutations cause cardiac arrhythmia (Long QT syndrome (LQTS), type 3 and Brugada syndrome, respectively) but are not reported to cause neuromuscular symptoms. In contrast to *Scn4a*, *Scn5a* expression is sensitive to the innervation pattern of the myofibers with downregulation during postnatal development and strong upregulation when the adult myofiber is denervated (Awad et al., 2001; Carreras et al., 2021). The upregulation of *Scn5a* upon denervation results from the binding of the Gata4 transcription factor, itself upregulated following denervation, to the *Scn5a* promoter region, and from epigenetic regulations mediated by the H3K27ac and H3K4me3 histones (Carreras et al., 2021).

## Loss-Of-Function Mutations IN *SCN4A* and Muscle Weakness

### Dominantly-Inherited Mutations and Episodes of Flaccid Muscle Weakness in Hypokalemic Periodic Paralysis

Familial hypokalemic periodic paralysis (HypoPP) is a rare condition characterized by the episodic occurrence of moderate to severe muscle weakness, which may be focal or generalized, concomitantly to low blood K^+^ levels (<3,5 mEq/L). The attacks are usually triggered by rest after strenuous exercise, carbohydrate-rich meal, or stress. Each attack has a gradual onset over minutes and resolves spontaneously after few hours or days (Statland et al., 2018). Familial and non-familial forms of HypoPP exist. Non-familial forms are most often secondary to another physiological dysfunction such as thyrotoxicosis, hyperaldosteronism or nephropathic K^+^ loss. Familial forms are dominantly-inherited and typically develop in the first or second decade of life. Inter-critical electromyographic testing based on compound muscle APs (CMAP) recording demonstrates a decrease of CMAP amplitudes in response to a 5 min-long exercise, which may help to document muscle weakness between episodes (McManis et al., 1986; Fournier et al., 2004). The definitive diagnosis of HypoPP lies on genetic testing with mutations in the genes known to cause HypoPP: *CACNA1S*, which encodes the pore-forming subunit of the skeletal muscle voltage-gated calcium channel Ca_v_1.1 (HypoPP1), or *SCN4A* (HypoPP2) (Jurkat-Rott et al., 1994; Ptáček et al., 1994; Jurkat-Rott et al., 2000). Dominant-negative mutations in the *KCNJ2* gene, encoding the K^+^ channel Kir2.1, also cause primary HypoPP in Andersen-Tawil syndrome, a disorder clinically distinct from HypoPP1 and 2 since the neuromuscular phenotype is associated with cardiac arrhythmia and bone deformities (Plaster et al., 2001).

Fourteen *SCN4A* missense mutations are reported to cause HypoPP2. Eleven substitute the two arginine residues (R1 or R2) nearest the extracellular end of S4 segments in DI, DII and DIII and cause HypoPP2 that are dominantly-inherited (Figure 2 and Table 1). The exception are p.Arg1135His/Cys and p.Arg1451Leu. The p.Arg1135His/Cys mutations substitute the 3rd arginine residue of DIIIS4 and cause *de novo* (p.Arg1135His) or recessively-inherited (p.Arg1135Cys) HypoPP2 (Groome et al., 2014). P.Arg1451Leu substitutes the 1st arginine residue of DIVS4 and results in HypoPP2 and myotonia in one homozygous individual (Groome et al., 2014; Luo et al., 2018). HypoPP2 mutations that substitute R1 or R2 as well as p.Arg1135His/Cys, but not p.Arg1451Leu, promote a gating pore current (Sokolov et al., 2007; Jurkat-Rott et al., 2009; Wu et al., 2012). The gating pore current (also named omega current) is a cation-selective inward current activated by hyperpolarization through an aqueous pathway created by the neutralization of one of the two outermost positive charges of DIS4, DIIS4 or DIIIS4 in Na_v_1.4 (Gosselin-Badaroudine et al., 2012). This small inward current explains the paradoxical depolarization characteristics of HypoPP myofibers: they are more frequently depolarized than control myofibers (resting membrane potential (RMP) equal to −55 mV instead of −90 mV) in low extracellular K^+^ (Rüdel et al., 1984). To simplify complex pathophysiological mechanisms, hypokalemia would be induced by muscle K^+^ reuptake after exercise or in response to insulin or glucocorticoid (such as cortisol) secretion following carbohydrate-rich meals or stress, respectively. Hypokalemia would modify the cumulated activities of K^+^ (especially Kir2.1) and Cl^-^ channels, ATPase pumps and Na-K-2Cl (NKCC) transporters in myofibers (Jurkat-Rott et al., 2009; Wu et al., 2011; Mi et al., 2019). These changes would establish the net balance of no ionic current at the depolarized (−55 mV) value in HypoPP2 myofibers because of the gating pore current. Na_v_1.4 channels would then be mostly inactivated, and the myofibers not excitable (Rüdel et al., 1984; Struyk and Cannon, 2008; Jurkat-Rott et al., 2009). The HypoPP2 mutations could therefore be considered as dominant-negative, the gating pore current altering the central pore current of mutant and wild-type (WT) Na_v_1.4 channels.

**TABLE 1:** *SCN4A* mutations with loss-of-function effects on Na_v_1.4 gating behavior.

Disease (inheritance)	Mutation (amino acids)	Domain (interaction)	Gating effects					References
			Current density	Activation	Fast inactivation	Slow inactivation	Inward gating pore current*	
CM (r)	p.Arg104His	N-terminus	Null					Zaharieva et al. (2016)
CM (hypokinesia, r)	p.Met203Lys	DIS3	-	-	-	-		Zaharieva et al. (2016)
PP with hypokalemic episodes (h)	p.Ala204Glu	DIS3	-	+/-	+	+	-	Kokunai et al. (2018)
HypoPP2 (h)	p.Arg219Lys	DIS4	=	=	=	nd	+ (low)	Kubota et al. (2020)
HypoPP2 (d)	p.Arg222Trp/Gly	DIS4	-	-	+	+	+	Bayless-Edwards et al. (2018), Mannikko et al. (2018)
NDM (d) CM (r)	p.Arg225Trp	DIS4	-	-	=	+	nd	Lee et al. (2009), Zaharieva et al. (2016)
CMS (r)	p.Ser246Leu	DIS4S5	=	=	=	+/-		Tsujino et al. (2003)
CM (r)	p.delTyr307_Gly367	DIS5S6 (Na_v_β1)	nd (presumed null)					Mercier et al. (2017)
CM (r)	p.Cys375Arg	DIS5S6 (Na_v_β1)	Null					Gonorazky et al. (2017)
CM (hypokinesia, H, r)	p.Pro382Thr	DIS5S6 (Na_v_β1)	Null					Zaharieva et al. (2016)
CM (r)	p.Gln470X	DIS6-DIIS1	nd (presumed null)					Zaharieva et al. (2016)
HypoPP2 (d)	p.Arg669His/Gly	DIIS4	-	=	+	+	+	Kuzmenkin et al. (2002), Mi et al. (2014), Struyk et al. (2000)
HypoPP2 (d)	p.Arg672His	DIIS4	-	=	+	=	+	Kuzmenkin et al. (2002)
HypoPP2 (d)	p.Arg672Gly/Cys	DIIS4	-	-	+	+	+	Jurkat-Rott et al. (2000), Kuzmenkin et al. (2002), Mi et al. (2014)
HypoPP2 (d)	p.Arg672Ser	DIIS4	=	=	+	+	nd	Bendahhou et al. (2001)
Normo PP with corticosteroid-induced hypokalemic episodes (d)	p.Arg675Gly/Gln/Trp	DIIS4	= /-	=	=	-	+	Sokolov et al. (2008)
CM (hypokinesia and classical CM, r)	p.Ala1049ValfsX50	DIIIS1	nd (presumed null)					Zaharieva et al. (2016)
CMS (r)	p.Arg1059X	DIIIS1S2	nd					Elia et al. (2019)
CM (hypokinesia and classical CM, r)	p.Asp1069Asn	DIIIS2	=	-	-	-		Zaharieva et al. (2016)
CM (r)	p.Ser1120Leu	DIIIS3S4	nd					Mercier et al. (2017)
HypoPP2 (d)	p.Arg1129Gln	DIIIS4	nd					Weber et al. (1993)
HypoPP2 (d)	p.Arg1132Gln	DIIIS4	=	+	+	+	+	Carle et al. (2006), Francis et al. (2011)
HypoPP2 (h^$^)	p.Arg1135His	DIIIS4	=	-	+	+	+	Groome et al. (2014)
HypoPP2 (r) CM (r)	p.Arg1135Cys	DIIIS4	=	+/-	+	+	+	Groome et al. (2014), Zaharieva et al. (2016)
CM (H, r)	p.Arg1142Gln	DIIIS4	-	-	-	=	nd	Gonorazky et al. (2017), Sloth et al. (2018)
CM (r)	p.Cys1209Phe	DIIIS5S6	Null					Zaharieva et al. (2016)
CMS (r)	p.Val1442Glu	DIVS3S4	=	=	+	=		Tsujino et al. (2003)
NDM, PP with hypokalemic episodes (h^$^), HypoPP2 and myotonia (H), HyperPP (d)	p.Arg1451Leu	DIVS4	-	-	+	=	-	Luo et al. (2018), Poulin et al. (2018)
Glucocorticoid-induced hypoPP (h)	p.Arg1451Cys	DIVS4	-	-	+	+	-	Poulin et al. (2018)
CMS (H, r)	p.Arg1454Trp	DIVS4	=	=	+/-	+	-	Habbout et al. (2016)
CMS (H, r)	p.Arg1457His	DIVS4	=	=	+/-	+	nd	Arnold et al. (2015)
CMS (H, r)	p.Arg1460Trp	DIVS4	-	=	+/-	=	-	Elia et al. (2019)
NDM (d) CMS (r)	p.Arg1460Gln	DIVS4	-	=	+/-	=	-	Elia et al. (2019)
CM (hypokinesia, r)	p.Tyr1593X	DIVS6	nd (presumed null)					Zaharieva et al. (2016)
CMS (H, r)	p.Pro1650Leu	C-ter	-	=	=	=		Echaniz-Laguna et al. (2020)
CM (r)	p.His1782Glnfs65	C-ter (syntrophin)	=	=	=	=		Zaharieva et al. (2016)

PP: periodic paralysis; HypoPP(2): hypokalemic periodic paralysis (type 2); CMS: congenital myasthenic syndrome; NDM: non dystrophic myotonia; normoPP: normokalemic PP; (r) recessive; (d) dominant; (h) heterozygous mutation with unknown inheritance pattern; (H) mutation found in the homozygous state; (+) enhanced; (-) impaired; (+/-) GoF and LoF effects *in vitro*; (=) no change; nd: not determined; * investigated when hypokalemic episodes were reported.

HypoPP2 mutations causing gating pore current have also been reported to cause LoF effects on Na_v_1.4 gating in heterologous cell expression systems (Bayless-Edwards et al., 2018; Bendahhou et al., 2001; Groome et al., 2014; Jurkat-Rott et al., 2000; Mi et al., 2014; Struyk et al., 2000). The LoF changes include reduced Na^+^ current density and enhanced fast and slow inactivation (Table 1). Whether they participate to the loss of muscle force in HypoPP2 is an open question. One argument for the physiological significance of these LoF changes is the smaller amplitude and the slower rate of rise of muscle APs in HypoPP2 myofibers compared to control myofibers at normal (−90 mV) RMP (Jurkat-Rott et al., 2000; Groome et al., 2014; Bayless-Edwards et al., 2018). Moreover, introducing one HypoPP2 missense mutation (p.Arg669His) into the mouse *Scn4a* gene by homologous recombination reproduces LoF changes on muscle APs in addition to the pathogenic gating pore current (Wu et al., 2011). A second argument comes from recent reports of *SCN4A* missense mutations that do not induce gating pore current in individuals suffering from PP with hypokalemic episodes of muscle weakness (Kokunai et al., 2018; Luo et al., 2018; Poulin et al., 2018). Two individuals are heterozygous for one missense substitution (p.Ala204Glu, p.Arg1451Leu) whereas one is homozygous for one of these two substitutions (p.Arg1451Leu). The recurrence of p.Arg1451Leu in unrelated individuals underlines the link between the functional changes induced by this *SCN4A* mutation and hypokalemic episodes of paralysis. P.Ala204Glu and p.Arg1451Leu exert GoF effects with enhanced activation. They also exert LoF effects with reduced Na^+^ current density, slower activation kinetics, accelerated entry into fast inactivation and slower recovery from slow inactivation for mutant channels compared to WT channels (Kokunai et al., 2018; Luo et al., 2018). For one (p.Ala204Glu), LoF changes were more severe in low K^+^_ext_ with a depolarization shift of activation observed for the mutant but not the WT channels (Kokunai et al., 2018). Recently, a charge-retaining substitution in DIS4 (p.Arg219Lys) has been reported to cause HypoPP2 by a mechanism distinct from gating pore current as the latter was too small to be predicted pathogenic (Kubota et al., 2020). Current density, activation and inactivation of Na_v_1.4 were unaffected by p.Arg219Lys, but slow inactivation was not investigated. If these works demonstrate that Na_v_1.4 LoF may result in hypokalemic episodes of muscle paralysis, it cannot be excluded that other genetics and/or environmental factors are important for their expressivity since they were not observed in familial forms.

### Recessively-Inherited Loss-of-Function Mutations in Sodium Channel Weakness

The demonstration that Na_v_1.4 LoF causes loss of muscle force has been provided in the last 5 years with the identification and the functional investigation of recessively-inherited *SCN4A* mutations in children with two forms of congenital muscle weakness: congenital myasthenic syndrome (CMS) and congenital myopathy (CM). Twenty-three recessively-inherited LoF mutations are now described in *SCN4A* (Figure 2 and Table 1). In contrast to the situation observed for dominantly-inherited Na_v_1.4 channelopathies, no recurrent *SCN4A* mutations have been described for recessively-inherited Na_v_1.4 channelopathies, the reported mutations being private. Both hypomorph (that causes a partial LoF) and null (that causes a complete LoF) mutations are observed.

**Congenital myasthenic syndromes** (CMS [MIM#608931]) form a clinically and genetically heterogeneous group of inherited disorders with skeletal muscle weakness that worsens with physical exertion (Engel et al., 2015; Nicole et al., 2017). They are caused by defective neurotransmission at the NMJ due to presynaptic (nerve terminal), synaptic cleft or most frequently post-synaptic (muscle) defects. Neurotransmission defects are documented by electromyographic testing showing a decrement of CMAP in response to repetitive nerve stimulation (RNS) at low (3 Hz) frequency or by single fiber electromyography (SFEMG) testing, a technique that detects neuromuscular transmission failure in motor units (Engel et al., 2015; Nicole et al., 2017). Thirty genes are known to cause CMS, which encode proteins that are critical for the formation, the maturation, the maintenance or the function of the NMJ such as post-synaptic nAChRs or proteins required for the synaptic aggregation of these receptors (agrin, Dok-7, rapsyn, MuSK, … ). CMS are most frequently recessively-inherited but dominant forms exist such as the slow-channel CMS that result from GoF mutations in the genes encoding nAChR subunits.

The first report of *SCN4A* mutations as the cause of fatigable muscle weakness suggestive of CMS was published nearly 20 years ago but the inheritance pattern (dominant or recessive) could not be established (Tsujino et al., 2003). This work reports one individual with abrupt paralytic attacks of respiratory and bulbar muscles in the neonatal period and general muscle weakness worsened by activity later in life. The patient was heterozygous for two *SCN4A* missense mutations (p.Ser246Leu and p.Val1442Glu). Electromyographic testing showed CMAP decrement at high (10 and 50 Hz) but not at low (2 Hz) RNS. One substitution (p.Val1442Glu) has strong LoF effects with enhanced fast inactivation, predicting that only 13% of Na_v_1.4 channels are available for activation at the RMP. The second substitution (p.Ser246Leu) was concluded to be benign with mixed enhancing and impairing effects on slow inactivation. The inheritance pattern of the disease remained therefore unclear. The existence of recessively-inherited *SCN4A* mutations as the cause of fatigable muscle weakness has indeed been proven only recently. Four original articles have reported eight homozygous or compound heterozygous missense mutations in six unrelated individuals (Arnold et al., 2015; Habbout et al., 2016; Elia et al., 2019; Echaniz-Laguna et al., 2020). Symptoms suggestive of CMS with a decrement of CMAP at high (10–50 Hz; 2 out 6 patients tested) but not at low (3 Hz) RNS and jitter at SFEMG (2 out 2) were observed. They were combined to symptoms of PP, CM or even myotonia for p.Arg1460 substitutions. The decrement of CMAP in response to the long-exercise test was reported for 4 out 5 patients, and mild fiber size variabilities on muscle biopsies were observed for 2 out 4 examined.

**FIGURE 4:**
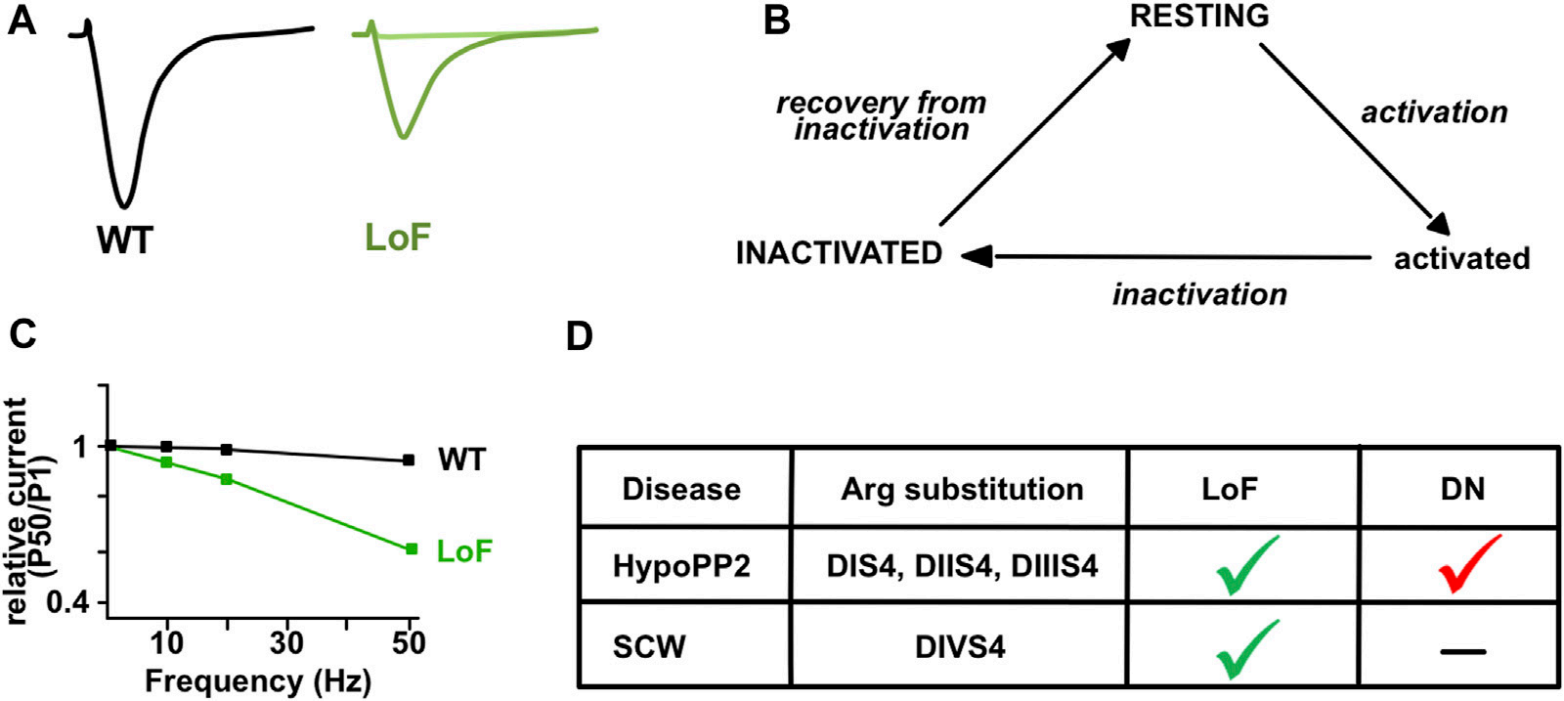
Gating of Na_v_1.4 with sodium channel weakness (SCW) mutations. **(A)** Illustrative Na^+^ current traces for wild-type (WT, black) and mutant (LoF, green) Na_v_1.4 channels obtained in HEK-293 cells. Differences include reduced current density, impaired activation, and enhanced inactivation for hypomorph mutants (dark green). No current is recorded for null mutants (light green). **(B)** Three-states model of Na_v_1.4 channels. The resting or inactivated states are favored in SCW. **(C)** Relative Na^+^ current amplitude (pulse 50 compared to pulse 1) is observed in response to pulse trains at 10, 20 and 50 Hz for the mutant (LoF, green) but not the WT (black) channels in heterologous cells (adapted from Habbout et al., 2016). **(D)** Main differences between dominantly-inherited HypoPP2 mutations and recessively-inherited SCW mutations substituting Arginine (Arg) residues in S4 segments of Na_v_1.4. The gating pore current induces a dominant-negative (DN) effect on central Na_v_ currents in HypoPP2. Loss of function (LoF) occurs in both.

Six of the eight *SCN4A* substitutions resulting in CMS are in or close to DIVS4, suggesting that this segment may be a hot-spot for *SCN4A* mutations causing fatigable muscle weakness (Figure 2). Four neutralize positively-charged residues and exert mixed GoF and LoF effects on fast inactivation (Table 1). They do not induce gating pore current in accordance with previous studies showing that the substitution of a single positively-charged residue in DIVS4 does not induce this current (Gosselin-Badaroudine et al., 2012). All the mutations investigated in patch-clamp experiments are LoF with reduced Na^+^ current density and/or enhanced fast or slow inactivation (Figure 4). Repetitive pulses at physiological frequencies (10–80 Hz) on heterologous cells induce a decrease of Na^+^ current amplitude for p.Val1442Glu, p.Arg1454Trp and p.Arg1457His mutant channels but not for WT or p.Ser246Leu channels (Figure 4) (Tsujino et al., 2003; Arnold et al., 2015; Habbout et al., 2016). Decrease of Na^+^ current in response to pulse trains (60–100 Hz) was on the contrary less pronounced for p.Arg1460Trp/Gln mutant channels than for WT channels (Elia et al., 2019). This may be due to the GoF effects of these variations, and may account for myotonia in individuals heterozygous for p.Arg1460Trp/Gln.

**Congenital myopathies (CM)** are another group of genetically and clinically heterogeneous diseases with muscle weakness (Gonorazky et al., 2018). There are many different types of CM with more than 30 known causative genes required for skeletal muscle development, structure or function. Most share common features, including lack of muscle tone and weakness. The degree of severity is wide, ranging from severe muscle weakness with *in utero* or neonatal lethality, to infant- or childhood-onset weakness (Wallgren-Pettersson and Laing, 2010). Several tests are performed to diagnose CM, including blood tests, electromyography, muscle biopsy and genetic testing. Fifteen recessive *SCN4A* mutations have been reported in nine unrelated kindreds with various severities of muscle weakness ranging from fetal hypokinesia (decreased or absent movements *in utero*) lethal at birth to “classical” CM for 11 individuals (Zaharieva et al., 2016; Gonorazky et al., 2017; Mercier et al., 2017; Sloth et al., 2018). Electromyographic investigations showed myopathic changes for some but not all individuals with classical CM (5 out 7). Marked fatigability with CMAP decrement at high (10Hz; 1 out 2 patients tested) but not at low (3 Hz) RNS was reported for some patients with “classical” CM (Zaharieva et al., 2016). When done, muscle biopsies showed fiber size variability, some necrotic myofibers and increased nuclear internalization but no specific structural abnormalities.

Mutations causing CM are observed on the entire length of Na_v_1.4 (Figure 2; Table 1). Four are supposed to be null as they delete several segments. Four missense substitutions (p.Arg104His, p.Cys375Arg, p.Pro382Thr, p.Cys1209Phe) are functionally null as they do not produce any Na^+^ current in heterologous cells. Two are in one (DIS5S6) binding site for β1 subunit. Whether these missense mutations are null because of defective membrane trafficking or abnormal gating of the mutant channel is unknown. Four substitutions are hypomorph since mutant channels are still functional but have greatly reduced Na^+^ current density or impaired activation. One (p.Arg1135Cys) is reported to cause a recessively-inherited HypoPP2 phenotype and a gating pore current *in vitro* (Groome et al., 2014). Another mutation (p.Arg225Trp) has been reported to be heterozygous in one Korean patient with SCM but does not display GoF characteristics *in vitro* (Lee et al., 2009). One frameshift mutation (p.His1782Glnfs65) does not induce any gating defect in heterologous cells*.* This frameshift mutation results in the loss of the syntrophin binding site in the C-terminal tail of Na_v_1.4 and its effect on the membrane expression of Na_v_1.4 in myofibers remains to be determined. That strengthens the important notion that Na_v_1.4 gating defects *in vitro* do not always fit the phenotypic expression. Moreover, the *in vitro* effects of pulse trains on Na^+^ current amplitude has not been tested for the *SCN4A* mutations causing CM.

**FIGURE 5:**
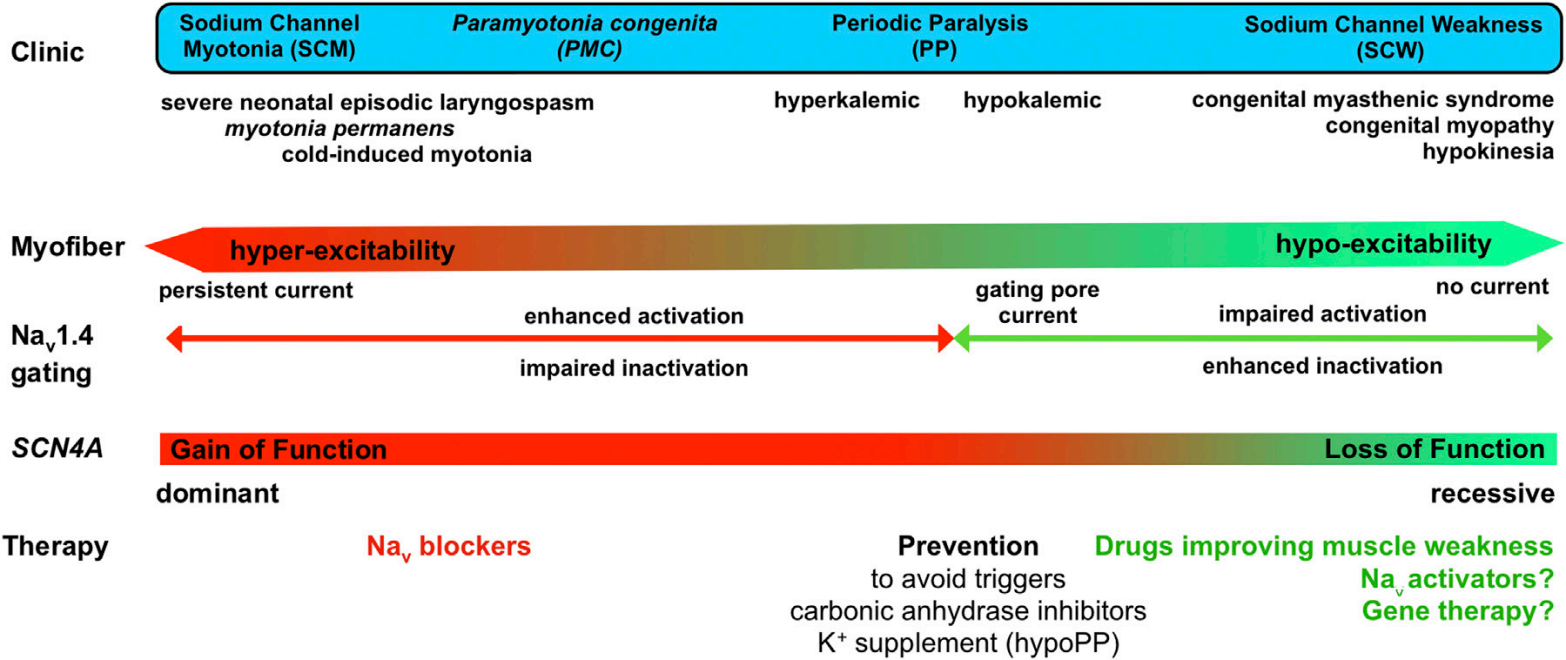
Clinical spectrum of Na_v_1.4 channelopathies with a continuum from membrane hyperexcitability in sodium channel myotonia (SCM) to hypoexcitability in sodium channel weakness (SCW), effect of the mutations on Na_v_1.4 gating and available as well as potential (?) therapeutic options.

**Sodium Channel Weakness (SCW)**. LoF mutations that favor non-conducting (resting or inactivated) states of Na_v_1.4 result in CMS, CM, and unusual forms of PP with hypokalemic episodes that are not due to a gating pore current (Figure 4). If PP, CMS and CM are distinct muscle disorders, the overlapping symptoms observed for individuals with Na_v_1.4 LoF mutations suggest that they form a clinical spectrum with a continuum of membrane hypoexcitability as proposed for Na_v_1.4 channelopathies due to GoF mutations (Figure 5) (Cannon, 2018). We propose here to name this spectrum “Sodium Channel Weakness (SCW)” as the clinical mirror of Sodium Channel Myotonia (SCM). A dosage-effect could occur in SCW that would explain the variable severity of muscle weakness: the fewer Na_v_1.4 would be functional, the more severe would be the muscle weakness. Additional individuals with Na_v_1.4 LoF mutations must be reported to ascertain this hypothesis important for genetic counseling and therapeutic issues. No specific biomarker (clinical signs, electromyographic testing or muscle biopsies investigations) has been identified for SCW, and the diagnosis of congenital muscle weakness due to Nav1.4 dysfunction requires to perform sequencing of *SCN4A* and to identify pathogenic variations.

One argument in favor of the dosage-effect hypothesis for SCW is the phenotype of *Scn4a* knock-out mouse mutants (Wu et al., 2016). Homozygous knock-out mice die at birth. Heterozygous mice have no gross locomotor deficits despite a half-reduced TTX-sensitive Na^+^ current recorded in dissociated myofibers. Loss of muscle force in response to factors inducing episodes of muscle weakness in PP was not observed in myofibers of heterozygous *Scn4a* ^*+/-*^ mice despite the reduced Na^+^ current (Wu et al., 2016). *Scn4a* haploinsufficiency has nevertheless a functional significance: if the tetanic force developed in response to supramaximal stimulus is like controls, a higher stimulating current density is required to achieve 50% maximal force in *Scn4a* ^*+/-*^ myofibers. Moreover, force decline is observed for *Scn4a* ^*+/-*^ myofibers in response to submaximal stimuli with higher sensitivity to partial blockade of neuromuscular transmission with curare (an antagonist of nAChR) compared to controls (Wu et al., 2016). This physiological work demonstrates that a half-reduction of functional Na_v_1.4 channels in myofibers is not sufficient to induce PP by itself but reduces muscle force in challenging conditions. More recently, the lack of Na_v_ channels clustering at the NMJ in mice knocked-out for ankyrins has also been reported to cause muscle weakness with reduced spontaneous locomotor activity of homozygous mutant mice and CMAP decrements in response to 40 and 60 Hz RNS (Zhang et al., 2021). These two elegant studies confirm that the lack of synaptic Na_v_1.4 or a half reduction in the whole amount of Na_v_1.4 in myofibers results in muscle weakness with a fatigability component (Wu et al., 2016; Zhang et al., 2021).

**FIGURE 6:**
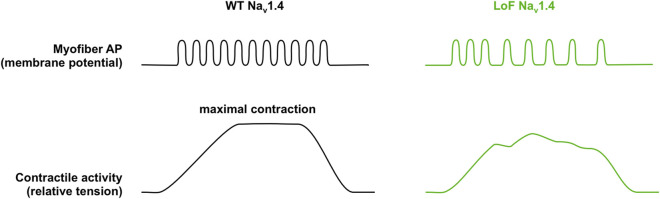
Schematic representation of a simple pathophysiological hypothesis to account for sodium channel weakness (SCW) due to Na_v_1.4 loss of function (LoF). Repetitive muscle action potentials (myofiber AP) resulting from motoneuronal firing lead to the summation of muscle twitches (contractile activity), in this example up to the maximal sustained contraction (tetanos) in wild-type muscles (WT Na_v_1.4, black traces). Na_v_1.4 LoF would reduce the amount of Na_v_1.4 channels available for activation. This would result in lower muscle AP frequency and muscle force in response to neuronal firing in mutant myofibers (LoF Na_v_1.4, green). The fatigability would result from a decrease of Na_v_1.4 availability during neuronal firing, which would progressively reduce muscle AP frequency and muscle force.

Altogether, these studies suggest the following simplistic pathophysiological mechanism to account for muscle weakness when Na_v_1.4 is deficient (Figure 6): LoF mutations would reduce the amount of Na_v_1.4 channels available for activation by reducing their quantity or by altering their biophysical properties. Consequently, the genesis of muscle APs in response to epp and/or their propagation along the sarcolemma would fail more frequently than normal. This would result in a lower muscle AP frequency in the transverse tubules and so in a lower muscle force in response to motoneuronal firing. A fatigable component of the muscle weakness would result, at least in part, from enhanced inactivation properties that reduce Na_v_1.4 availability for activation with repetitive stimulations.

### *SCN4A* Variants, Susceptibility to Sudden Infant Death Syndrome and Importance of Modifying Factors

To complete this overview of Na_v_1.4 LoF in human diseases, a case-control genetic study reports more frequent *SCN4A* missense variants in infants deceased from sudden infant death syndrome (SIDS) than in controls (Mannikko et al., 2018). SIDS is a sudden, unexpected and unexplained death of an apparently healthy baby. The exact cause of SIDS is unknown and is thought to result from a combination of factors. Among the six *SCN4A* variants observed in a SIDS sample of 278 infants, four have a functional impact on Na_v_1.4 current: two are GoF and two are LoF (p.Val1442Met in DIVS3S4 and p.Glu1520Lys in DIVS5S6). One (p.Val1442Met) substitutes one residue that induces SCW when substituted by Glu. An independent study performed in another sample of 73 infants with SIDS reported two inherited heterozygous missense variants (p.Lys724Arg in DIIS5S6 and p.Phe103Val in N-terminus) in *SCN4A* in two infants, but the biophysical characterization of the variant channels has yet to be done (Rochtus et al., 2020). The size of the two studied SIDS samples is low, and robust replicative studies are required to confirm Na_v_1.4 dysfunction as a susceptibility factor for SIDS. In addition, several challenges are raised by these genetic association studies. The first is that the variant effects should lead to deleterious function *in vivo*. The second is the question of identifying infants with *SCN4A* variants and at risk for SIDS with all the bioethical issues raised by genetic testing.

These genetic association studies and the identification of Na_v_1.4 LoF mutations as the cause of a large clinical spectrum of congenital muscle weakness further underline the influence of modifying factors on the phenotypic expression of *SCN4A* mutations already reported for GoF mutations (Nicole and Fontaine, 2015). For example, the p.Arg1451Leu substitution results in NDM (cold-induced myotonia), HyperPP, dominant PP with hypokalemic episodes or HypoPP2 with myotonia when homozygous, whereas p.Arg1135Cys results in recessive HypoPP2 or CM (Table 1) (Groome et al., 2014; Zaharieva et al., 2016; Luo et al., 2018; Poulin et al., 2018). The exact identity of the modifying factors is not known, although knowledge of muscle physiology makes ion channels (including *trans*- and *cis*- *SCN4A* variants), proteins important for the fine-tuning of Na_v_1.4 channels and environmental factors strong candidates.

## Therapeutic Options for Sodium Channel Weakness due to Na_V_1.4 Loss of Function

### Use of Treatments Available for Na_v_1.4 Channelopathies and Congenital Myasthenic Syndromes

Several drugs are available as therapeutic tools against Na_v_1.4 GoF for clinical use in humans (Jitpimolmard et al., 2020; Desaphy et al., 2021). Use-dependent Na_v_ blockers are efficient to reduce myofiber hyperexcitability and improve myotonic symptoms. The first line blocker is mexiletine with conclusive clinical trials and European orphan drug designation (Statland, 2012; Stunnenberg et al., 2018b). Therapeutic management of PP mostly relies on patient education with a life-style aimed to avoid triggering factors. Carbonic anhydrase inhibitors (acetazolamide and dichlorphenamide) also help to prevent paralytic episodes in PP, in addition to K^+^ supplement in HypoPP. Carbonic anhydrase inhibitors are known to prevent and improve attacks in HypoPP since several years but their mode of action is unclear. Acetazolamide is a diuretic drug used to treat several illnesses. It modulates multiple processes by inducing acidosis and by activating ion channels such as Ca^2+^-activated K^+^ (BK) and Cl^−^ channels, and probably acts by this means in PP (Eguchi et al., 2006; Tricarico et al., 2006). Bumetanide, an antagonist of NKCC, is also efficient to prevent hypokalemic-induced weakness in HypoPP1 and 2 mice (Wu et al., 2013a; Wu et al., 2013b). It acts by limiting the intracellular Cl^−^ concentration rise during repetitive APs, which helps to maintain the HypoPP fibers in the normal RMP when extracellular K^+^ concentration is low. Unfortunately, bumetanide was not efficient in a randomized controlled trial (RCT) pilot study performed on 10 individuals with HypoPP, a negative result that might be due to the small size of the sample. Molecules are also available to improve muscle force in CMS by modulating neuromuscular transmission (Engel, 2018). They include K^+^ channel blockers (3,4-diaminopyridine), acetylcholinesterase inhibitors (pyridostigmine), agonists of β2 adrenergic receptors (albuterol, ephedrine) for most forms of CMS and blockers of nAChRs for slow-channel CMS (quinidine, quinine, fluoxetine). No generic or specific treatments are available for CM, although several specific, especially gene-based, therapies are either in preclinical development or in early trials (Wallgren-Pettersson and Laing, 2010; Gonorazky et al., 2018).

Some but unfortunately not all individuals with SCW due to hypomorph *SCN4A* mutations benefited from pyridostigmine with acetazolamide or from acetazolamide alone (Arnold et al., 2015; Habbout et al., 2016; Elia et al., 2019; Echaniz-Laguna et al., 2020). Contrary to severe Na_v_1.4 GoF conditions that greatly benefit from Na_v_ blockers (mexiletine, carbamazepine), there is no treatment reported to prevent early death linked to *SCN4A* LoF mutations in SCW (Gay et al., 2008; Lion-Francois et al., 2010).

### Possible Therapeutic Strategies to Improve Muscle Force When Na_v_1.4 Is Deficient

Several inherited Na_v_ channelopathies are caused by LoF mutations such as Brugada syndrome, a cardiac arrhythmia due in part to Na_v_1.5 gating defects, and Dravet syndrome, a severe and lifelong form of pediatric epilepsy due to Na_v_1.1 haploinsufficiency (Loussouarn et al., 2015; Mantegazza et al., 2021). In all, a dosage effect is suggested, driving the therapeutic effort on boosting the mutant Na_v_ channels to eventually improve the clinical consequences of their LoF. The hypomorph nature of several *SCN4A* LoF mutations and the dosage effect suspected to occur in SCW suggest that such an approach would also be efficient for Na_v_1.4 channelopathies (Figure 5).

Detailed pharmacological characterizations of Na_v_ activators are sparse compared to inhibitors and the current challenge fostered by the existence of LoF Na^+^ channelopathies is to identify Na_v_ channels agonists selective enough to be clinically useful. A complementary strategy to target-based drug approach would be to perform phenotype screening in order to identify molecules able to improve muscle weakness when Na_v_1.4 is deficient (Swinney and Lee, 2020). The last few years have seen the impressive development of gene-based therapy for monogenic diseases with efficient vectors for selective gene delivery *in vivo* and the use of CRISPR-Cas9 technology for genome editing. Promising preclinical results have been obtained for some Na_v_ channelopathies, which could eventually benefit Na_v_1.4 channelopathies.

#### Target-Based Drug Screening to Identify Na_v_1.4 Activators

The goal of this approach is to identify molecules that will preferentially or selectively activate Na_v_1.4 channels. Na_v_ agonists may act by facilitating activation or by impairing inactivation (Figure 4) (Deuis et al., 2017). Different classes of Na_v_ activators are known, including pyrethroid insecticides and natural compounds such as alkaloid-based or peptide toxins from venoms. The high sequence conservation of Na_v_ channels renders challenging the identification of highly subtype-specific modulators, but this is nevertheless possible. For example, AA43279 is one small chemically synthetized compound identified in a screening campaign looking for Na_v_1.1 activators. AA43279 acts as an inactivation blocker and appears efficient to functionally counteract Na_v_1.1 haploinsufficiency in a zebrafish model of Dravet syndrome (Frederiksen et al., 2017; Weuring et al., 2020). Due to their serious physiological effects and co-evolution of preys and predators, several Na_v_ modulators including isoform-specific activators, are found in a variety of animal samples. Interestingly, amino acid substitutions may drastically change the toxin affinity for Na_v_ isoforms. This is well exemplified by the natural resistance of poison frog Na_v_1.4 to alkaloids such as batrachotoxin—a use-dependent activator produced by *Phyllobates* poison dart frog—to prevent self-intoxication (Tarvin et al., 2016). Some Na_v_ agonists have been successfully used in preclinical studies, rendering peptide toxins useful drug leads for developing novel therapeutic agents. For example, the spider-venom peptide Hm1a binds DIVS1S2 and DIVS3S4 extracellular loops of Na_v_ channels and specifically activates Na_v_1.1 and Na_v_1.3 isoforms by inhibiting the VSD gating movement of DIV and hindering both fast and slow inactivation (Osteen et al., 2017; Osteen et al., 2016). Hm1a leads to increased Na_v_ channels availability during high-frequency stimulations and its intracerebroventicular infusion reduced the Dravet syndrome-like phenotype of mice with Na_v_1.1 haploinsufficiency (Osteen et al., 2016; Richards et al., 2018).

A few Na_v_ channel activators are known to have a great affinity for Na_v_1.4 such as the sea anemone AFT-II, the α-scorpion toxin OD-1 and the β-scorpion toxins Css4 and Tz1 (Table 2) (Deuis et al., 2017). AFT-II (for *Anthopleura fuscoviridis* anemone) is a 48-amino acid-long peptide with three disulfide bonds that preferentially binds Na_v_1.4 and Na_v_1.5 (Oliveira et al., 2004). It slows the inactivation process with depolarized shift and increased time constants, but its effect on cell excitability has not been explored. OD-1 is an amidated polypeptide of 65 amino acids with four disulfide bonds that efficiently binds Na_v_1.4, Na_v_1.6 and Na_v_1.7 but not Na_v_1.2, Na_v_1.3 or Na_v_1.5 (Durek et al., 2013). The agonist activity of purified OD-1 on Na_v_1.4 relies on hyperpolarizing shift of activation (Table 2). The *Odontobuthus doriae* venom, from which OD-1 has been isolated, causes pre- and post-synaptic repetitive firing in response to a single stimulus in mouse nerve-muscle preparations (Jalali et al., 2007). In agreement with its potent agonist effect, OD-1 increases Na^+^ current peak amplitude and neuronal AP frequency and decreases neuronal AP threshold on rat hippocampal brain slices, but its effect on nerve muscle preparations has not been studied (Lai et al., 2020). Another interesting toxin to explore for SCW is Css4 (for *Centruroides suffusus*). Css4 is a 65-amino acid-long β-scorpion toxin that activates Na_v_ channels by enhancing activation with an hyperpolarizing shift of its voltage dependence (Cohen et al., 2007). If Css4 is a Na_v_1.4 agonist, it has no effect on Na_v_1.5 activation, and substituting three amino acids of Css4 further increases its selectivity for Na_v_1.4. Interestingly, a genetically-modified form of Css4 counteracts the LoF effects of the HypoPP2 p.Arg672Gly mutation *in vitro* (Cohen et al., 2007). This modified toxin could be preclinically tested in the knock-in mouse model available for HypoPP2 to determine whether Na_v_1.4 LoF has a physiological significance in this form of SCW (Wu et al., 2011). Tz1 (for *Tityus zulianus*) is another β-scorpion toxin (63 amino acids) that induces a hyperpolarized shift of Na_v_ activation with high specificity to Na_v_1.4 and no effect on Na_v_1.7 or Na_v_1.5 (Leipold et al., 2006). Unfortunately, β-scorpion toxins, including Css4 and Tz1, have a bimodal effect on a use-dependent basis since they may reduce Na_v_ conductance, thereby depressing cell excitability. For example, Tz1 reduces Na_v_1.4 current at 0.1 Hz but increases it at 2 Hz (Cestèle et al., 1998; Leipold et al., 2012). Moreover, its antagonist, but not its agonist, effect is observed on Na_v_1.5 current at both frequencies (Leipold et al., 2012). Calliotoxin (δ-elapitoxin-Cb1a) is a 57-amino acid-long three-finger peptide from the *Calliophis bivirgatus* snake. It increases muscle force in chicken nerve-muscle preparations, and enhances peak of Na_V_1.4 current, shifts the voltage-dependence of its activation to more hyperpolarized potentials, delays its inactivation and causes a persistent Na^+^ current in heterologous cell expression systems (Yang et al., 2016). However, the activity and selectivity of calliotoxin have not been tested among Na_v_ channels and the boosting effect of this peptide on muscle force may be also due to activation of neuronal Na_v_ channels. Although not natural, 16-amino acids-long peptides corresponding to DI, DII and DIIIS4S5 intracellular linkers increase Na^+^ current density and shift hNa_v_1.4 activation towards hyperpolarization by allosterically modulating the activation gate and stabilizing the open state (Malak et al., 2020). These synthetic peptides add to the natural toxin arsenal towards identifying compounds able to selectively boost Na_v_1.4 activity.

**TABLE 2:** Some Na_v_1.4 activators reported in the literature. Their effect on Na_v_1.4 gating behavior *in vitro* is stated.

Compound	Nature	Size (amino acids)	Mode of action on Na_v_1.4	References
AFT-II	sea anemone toxin	48	depolarized shift of inactivation; increased time constants of inactivation	Oliveira et al. (2004)
OD-1	α-scorpion toxin	65	hyperpolarized shift of activation	Durek et al. (2013)
Css4	β-scorpion toxin	65	hyperpolarized shift of activation; enhanced closed state inactivation	Cohen et al. (2007)
Tz1	β-scorpion toxin	63	hyperpolarized shift of activation; slowed activation (at 0.1 Hz)	Leipold et al. (2006)
Calliotoxin	snake toxin	57	hyperpolarized activation; slowed inactivation	Yang et al. (2016)
DIS4S5, DIIS4S5, DIIIS4S5 linkers	synthetic peptides	16	hyperpolarized activation; stabilized open state	Malak et al. (2020)

If natural toxins and synthetic peptides are promising lead compounds, extensive preclinical research efforts are required to determine whether they may used for developing peptide-based therapeutics to counteract Na_v_1.4 LoF and improve SCW (Peigneur and Tytgat, 2018). Besides improving Na_v_1.4 specificity, rational control of the bimodal activity observed for some toxins is necessary to increase and not reduce myofiber excitability. Recent technological breakthroughs will foster these studies, especially CryoEM by facilitating structural pharmacology and the design of Na_v_ subtype-selective profiles (Pan X. et al., 2018; Kong et al., 2019; Noreng et al., 2021). Automated patch-clamp approaches will also help identify additional candidates by performing medium-throughput screening campaigns of toxin and chemical libraries (Pan J. Q. et al., 2018; Obergrussberger et al., 2018). Important preclinical steps for all Na_v_1.4 activators will be to determine whether they are able 1) to restore a ‘normal’ Na_v_1.4 current from mutant channels *in vitro*; 2) to improve muscle force without inducing myotonia or paralysis in preclinical animal models; and 3) are safe enough to be used in humans. These druggability parameters are clues for a therapeutic future of any molecule found to activate Na_v_1.4 *in vitro*.

#### Phenotypic Drug Screening to Reduce the Physiological Impact of Na_v_1.4 Loss of Function on Muscle Force

The benefic effect of pyridostigmine and acetazolamide reported for some individuals with SCW illustrates the rationale of such an approach (Tsujino et al., 2003; Habbout et al., 2016; Elia et al., 2019). Phenotypic drug screening has also been fruitful to identify promising lead compounds for other Na_v_ channelopathies. By screening a Food and Drug Administration (FDA)-approved compounds library, clemizole has been identified as efficient to improve the epileptic phenotype resulting from Na_v_1.1 haploinsufficiency on a zebrafish model for Dravet syndrome (Baraban et al., 2013). Based on the fact that Na_v_1.6 GoF results in severe epileptic phenotypes, two Na_v_1.6 inhibitors have also been shown to improve the phenotype in another zebrafish model of Dravet syndrome with Na_v_1.1 haploinsufficiency (Weuring et al., 2020).

These examples underline the notion that preclinical animal models of SCW are required to successfully perform phenotype drug screening using muscle weakness as a biomarker, as well as for the preclinical investigations of Na_v_1.4 activators listed above. Some animal models have been developed for Na_v_1.4 GoF: knock-in mouse lines with HyperPP or HypoPP2, and transgenic zebrafish lines with NDM (Hayward et al., 2008; Wu et al., 2011; Nam et al., 2019). No viable animal model with SCW due to *Scn4a* LoF has been reported yet. Mice homozygous for a null *Scn4a* allele die at birth, preventing their use as an efficient model for screening. In the zebrafish, which is a versatile vertebrae model for phenotypic drug screening of small molecule libraries, two *Scn4a* orthologs exist and are expressed in skeletal muscles (Novak et al., 2006; Chopra et al., 2010). This greatly complexifies the establishment of mutant lines with recessively-inherited *Scn4a* mutations in this small vertebrate.

The ongoing development of neuromuscular organoids using human induced pluripotent stem cells is another exciting possibility to preclinically model Na_v_1.4 LoF (Faustino Martins et al., 2020; Mazaleyrat et al., 2020)**.** *SCN4A* is expressed in the neuromuscular organoids obtained so far, but its contribution to Na^+^ current and muscle cell excitability must be explored. In addition, the current protocols of differentiation and 3D cell culture have to be further improved since they do not result in NMJ mature enough to be structurally and functionally relevant (Lynch et al., 2021). Such cellular models are nevertheless very promising as they should help deciphering, in patient-derived neuromuscular preparations, the impact of Na_v_1.4 mutations on excitability and contractility of innervated myofibers and testing novel (bio)pharmacological strategies in Na_v_1.4 channelopathies.

#### The Potential of Gene-Based Strategies

Gene-based therapies are now biomedical options for devastating monogenic diseases with the development of antisense oligonucleotides (ASO) and recombinant adeno-associated virus (rAAV). More recently, the breakthrough allowed by CRISPR-Cas9 technology has opened a gigantic new area in the field of therapies based on genome engineering. Promising preclinical results using these technologies have been obtained for Na_v_1.1 and Na_v_1.5 channelopathies. An ASO-based strategy has been successfully used to enhance the expression of the WT *Scn1a* allele in a mouse model of Dravet syndrome in order to circumvent Na_v_1.1 haploinsufficiency (Han et al., 2020). A CRISPR-Cas9 based strategy, which consists in positively and specifically modulating the expression of any gene of interest with a catalytically dead Cas9, has also been successfully used to promote *Scn1a* expression in mouse models of Dravet syndrome (Colasante et al., 2020; Yamagata et al., 2020). It would be interesting to use these technics to selectively boost *SCN5A* expression in skeletal myofibers and determine whether its overexpression may compensate Na_v_1.4 deficiency.

Efficient gene transduction is another way to compensate LoF mutations. AAV vectors are the vectors of choice for efficient gene transduction *in vivo* with good safety profile, lack of genome integration, long-term expression in non-dividing cells and tissue-specific tropism (Aguti et al., 2018). The length of the cDNAs coding for the α subunits of Na_v_ yet prevents their use, the maximal DNA size encapsulated by rAAV being smaller than 4.7 kb. Development of novel tools, such as *trans*-splicing events after splitting the coding sequence and packaging it into independent rAAVs, is therefore a prerequisite for efficient delivery of any cDNA of Na_v_ channels. A dual rAAV vector strategy promoting *trans-*splicing has been successfully used to overexpress one GoF variant of human *SCN5A* in mouse heart*,* opening the door to such an approach for efficiently expressing exogenous Na_v_1.4 in skeletal myofibers (Doisne et al., 2021).

Gene-based strategies are in full expansion with successful examples for devastating neuromuscular disorders such as spinal muscular atrophy with agreements from FDA and European Medical Agency for ASO-based and AAV-based gene therapies (Messina and Sframeli, 2020). We are nevertheless far away to use these approaches for SCW as it requires time to develop potent recombinant vectors and to accurately investigate their therapeutic potential and safety in preclinical models.

## Conclusion

These last 5 years have seen the identification of sodium channel weakness (SCW) as a subset of muscle channelopathies. A more complete and accurate clinical spectrum can now be drawn for Na_v_1.4 channelopathies with a full continuum of myofiber excitability ranging from hyperexcitability in SCM due to GoF changes to unexcitable myofibers in SCW due to LoF changes (Figure 5). Within this spectrum, the quality of life of patients is greatly reduced, and the extreme cases are life-threatening. The identification of LoF mutations in SCW strengthens the need for therapeutic tools able to counteract the loss of muscle force resulting from reduced or lack of Na_v_1.4 activity. The recent technological breakthroughs in omics technologies, medium-throughput patch-clamp procedures, 3D cultures, induced pluripotent stem cells, and gene-based therapies open exciting possibilities to fill these gaps for the eventual benefit of individuals with Na_v_1.4 channelopathies in the next years.
